# Optic neuritis in demyelinating diseases: study of 38 cases

**DOI:** 10.1055/s-0044-1792093

**Published:** 2024-12-15

**Authors:** João Marcos Campos Ferreira, Cristiane Rebello Gomes de Souza Fontes, Carolina do Val Ferreira Ramos, Osvaldo J. M. Nascimento

**Affiliations:** 1 Universidade Federal Fluminense, Faculdade de Medicina, Programa de Pós-graduação Stricto Sensu em Neurociências e Neurologia, Niterói RJ, Brazil.; 2 Instituto do Cérebro do Sul Fluminense, Volta Redonda RJ, Brazil.

**Keywords:** Optic Neuritis, Multiple Sclerosis, Neuromyelitis Optica, Myelin Sheath, Neurite Óptica, Esclerose Múltipla, Neuromielite Óptica, Bainha de Mielina

## Abstract

**Background** Optic neuritis is an inflammation of the optic nerve caused by genetic factors, external influences, and the activation of cross-reactive immune responses to infections.

**Objective** To describe the clinical and epidemiological characteristics of patients presenting optic neuritis as the initial symptom of some demyelinating diseases, divided among multiple sclerosis (MS), neuromyelitis optica spectrum disorder (NMOSD), and myelin oligodendrocyte glycoprotein-immunoglobulin G (MOG-IgG)-associated disorders (MOGADs).

**Methods** Thirty-eight patients who had optic neuritis as their first symptom and later developed MS, NMOSD, or MOGADs were analyzed.

**Results** There were thirty-four female patients (90%) and 4 male patients (10%); 23 (60%) were white and 15 (40%) were black. The most frequent definitive diagnosis was MS, with 24 (63%) cases, followed by NMOSD with 12 (32%) cases, and MOGADs with 2 (5%) cases. Regarding age, 9 (24%) were under 20 years old, 19 (50%) were between 20 and 30 years old, 6 (21%) were between 31 and 40 years old, and 2 (5%) were over 41 years old. As for the time to diagnosis, MS required 54 months in the public sector and 12 months in the private sector. Neuromyelitis optica spectrum disorder took 108 months in the private sector and 14.5 months in the public sector, while MOGADs averaged 2 months in the private sector.

**Conclusion** Patients with optic neuritis as the initial symptom were predominantly female, aged between 21 and 40 years, and of predominantly white ethnicity, with a higher prevalence of MS. Additionally, a direct relationship between the healthcare sector and the time to diagnosis became evident.

## Resumo

**Antecedentes** A neurite óptica é uma inflamação do nervo óptico desencadeada por diversos fatores, incluindo os aspectos genéticos, as influências externas e a ativação de resposta imune cruzada a infecções.

**Objetivo** Descrever as características clínicas e epidemiológicas dos pacientes que apresentaram neurite óptica como sintoma inicial de alguma doença desmielinizante, divididas entre esclerose múltipla (EM), distúrbio do espectro da neuromielite óptica (DENMO)e mielina oligodendrócito glicoproteína anticorpo associada à doença (MOGAD).

**Métodos** Trinta e oito pacientes que tiveram neurite óptica como primeiro sintoma e posteriormente EM, neuromielite óptica ou MOGAD) foram analisados.

**Resultados** Trinta e quatro pacientes eram do sexo feminino (90%), e 4, do sexo masculino (10%); 23 (60%) eram brancos, e 15 (40%), pretos. O diagnóstico definitivo mais frequente foi EM 24 (63%) casos, seguido DENMO 12 (32%) e MOGAD 2 (5%). Em relação à idade, 9 (24%) possuem idade inferior a 20 anos, 19 (50%) entre 20 e 30 anos, 6 (21%) entre 31 e 40 anos e 2 (5%) idade superior a 41 anos. Quanto ao tempo para diagnóstico, a EM necessitou de 54 meses no sistema público e no privado 12 meses, a DENMO 108 meses no setor privado e 14,5 meses no sistema público e a MOGAD levou uma média de 2 meses no setor privado.

**Conclusão** Conclui-se que pacientes que apresentaram neurite óptica como sintoma inicial são do sexo feminino, na faixa etária entre 21 e 40 anos, de etnia predominantemente branca, e tiveram maior prevalência de EM. Além disso, ficou clara a relação direta do setor de saúde com o tempo para o diagnóstico.

## INTRODUCTION

A growing area of study with notable development recently has been neuroimmunology, both in terms of diagnostic methods and treatments. The main clinical disorders involved in this area of neurology are multiple sclerosis (MS), neuromyelitis optica spectrum disorder (NMOSD), and myelin oligodendrocyte glycoprotein-immunoglobulin G (MOG-IgG)-associated disorders (MOGADs). These diseases are imbalances of the central nervous system of an inflammatory and degenerative nature, affecting young to middle-aged individuals and can lead to significant neurological sequelae with a substantial impact on the quality of life and work capacity of patients. 1 2 3 4

Characterized by their autoimmune etiology, demyelinating diseases (DDs) exhibit in their pathophysiology an attack to the myelin sheath, the substance that covers the axons in the central nervous system. This condition, exemplified by multiple sclerosis (MS), transverse myelitis, leukodystrophies and optic neuritis (ON), presents a wide range of clinical manifestations and temporal profiles of development, including the possibility of relapses. 5

Optic neuritis, an inflammation of the optic nerve, can be caused by various factors, including genetic aspects (a strong association with the HLA-DRB1 and HLA-B27 genes), external influences (trauma and/or toxins), and the activation of a cross-immune response to infections, which is the main mechanism. 6 7

Regarding the activation mechanism of the cross-immune response, it can occur with the reaction of serological markers of the type immunoglobulin G (IgG), including MOG-IgG, which targets the MOG glycoprotein in the myelin present in oligodendrocytes. Another influential immunoglobulin in the process is NMO-IgG, which reacts against the aquaporin-4 present in astrocytic cells. 5 7

Depending on the stimulus response to the neuronal structuring cells and the impairment of propagation of the nerve impulse from the retina to the lateral geniculate bodies, the possibility of visual impairments arises, presenting with the most typical symptoms of ON: ocular pain on movement with acute loss of unilateral visual acuity. In more atypical cases, symptoms such as disc or peripapillary hemorrhages and even optic nerve edema may occur. 8 9 10

The epidemiological data available regarding ON come from the prospective multi-center study called the Optic Neuritis Treatment Trial (ONTT). It is known that the pathology affects young adults aged between 30 and 35 years, with a greater predominance among females and Caucasians, with an incidence varying from 1 to 3:100,000. 7 11

Demyelinating disease is one of the most common etiologies of ON, especially MS. Today, it is known that around 50% of MS patients will present with ON, including the possibility of relapses. 10 An association measured by the ONTT study traces one of the most important points of the present research: after 10 years of follow-up from the onset of the ON condition, approximately 38% of the patients developed MS. This observation highlights the importance of recognizing ON as the first manifestation of a DD, recognizing its clinical-epidemiological profile. 8

The ON clinical presentation can vary across NMOSD, MS, and MOGAD. 12 Neuromyelitis optica spectrum disorder typically exhibits severe, unilateral vision loss progressing rapidly over days, linked with aquaporin-4 antibodies and a propensity for incomplete recovery and recurrent episodes. Conversely, MS presents with gradual unilateral vision loss over days to weeks, associated with multifocal central nervous system (CNS) lesions and varying degrees of recovery over weeks to months. In contrast, MOGAD showcases rapid-onset unilateral or bilateral vision loss akin to NMOSD, but it is characterized by antibodies against MOG, often resulting in favorable visual recovery, occasionally involving bilateral optic neuritis episodes. Understanding these nuanced differences in ON presentation across NMOSD, MS, and MOGAD is paramount for precise diagnosis and tailored management approaches. 12

Therefore, the present study aims to describe the clinical and epidemiological characteristics of patients who presented ON as the initial symptom of some DD, including MS, NMOSD and MOGAD, at the neuroimmunology outpatient clinic of Hospital Universitário Antônio Pedro and referred by collaborating neurologists and neuroophthalmologists (from the state of Rio de Janeiro).

## METHODS

The present work is a qualitative action research study aiming to investigate the clinical/epidemiological profile of ON, and it involves patients to better understand the epidemiology of this comorbidity. It is characterized as collaborative action research involving neurologists and their patients. This study was approved by the ethics committee under the number CAE 11529719.0.0000.5284.

The medical records of 38 patients of different ages and both sexes from the neuroimmunology outpatient clinic of Hospital Universitário Antônio Pedro and those referred by collaborating neurologists and neuro-ophthalmologists (from the state of Rio de Janeiro) from January 2021 to December 2021 were reviewed. Patients met the following inclusion criteria: a previous diagnosis of DD (MS, NMOSD, or MOGAD) respecting the diagnostic criteria of each (2017 McDonald's criteria for MS, the 2015 International Panel for NMO Diagnosis (IPND) for NMOSD [ Table 1 ], and in the case of MOGAD, a positive antibody by the CBA technique capable of measuring specific monoclonal antibody levels); and ON being the first symptom/outbreak of the disease. 12 13 Optic neuritis was considered in patients who presented a typical clinical condition as previously reported, associated or not with optic disc edema, pain and changes in magnetic resonance imaging (MRI). The exclusion criteria were patients who did not meet the necessary criteria for the diagnosis of MS, NMOSD, or MOGAD and patients who presented other neurological symptoms prior to ON or in cases of diagnostic doubt.

**Table 1:** The 2015 International Panel for NMO Diagnosis criteria for NMOSD

**Diagnostic criteria for NMOSD with AQP4-IgG**
At least 1 core clinical characteristic
Positive test for AQP4-IgG using best available detection method (cell-based assay strongly recommended)
Exclusion of alternative diagnoses
**Diagnostic criteria for NMOSD without AQP4-IgG or NMOSD with unknown AQP4-IgG status**
At least 2 core clinical characteristics occurring as a result of one or more clinical attacks and meeting all of the following requirements: At least 1 core clinical characteristic must be optic neuritis, acute myelitis with LETM, or area postrema syndrome Dissemination in space (2 or more different core clinical characteristics) Fulfillment of additional MRI requirements, as applicable
Negative tests for AQP4-IgG using the best available detection method, or testing
Exclusion of alternative diagnoses

Abbreviations: AQP4-|gG, aquaporin-4 immunoglobulin G antibody; LETM, longitudinally-extensive transverse myelitis; NMO, neuromyelitis optica; NMOSD, neuromyelitis optica spectrum disorder.

Note: Adapted from Wingerchuk et al., 2015. 13

The clinical and demographic characteristics were recorded in a database in a Microsoft Excel spreadsheet (Microsoft Corp, Redmond, WA, United States). In the data analysis, some variable factors were considered: age, gender, ethnicity (self-defined by the patients), comorbidities, access to health plans, type of neuritis (unilateral or bilateral), and time and form of neuritis diagnosis.

## RESULTS

Of the 38 patients, 34 were female (90%) and 23 (60%) were white. The most frequent definitive diagnosis was MS, with 24 (63%) cases, followed by NMOSD, with 12 (32%); all patients with NMOSD tested positive for aquaporin-4 antibodies using the CBA method, and MOGAD with 2 (5%). Regarding the age range of neuritis presentation, 19 (50%) patients were aged between 20 and 30 years ( Table 2 ).

**Table 2:** General description of the studied population

		Total of patients (n = 38; 100%)
**Sex**	**Female**	34 (90%)
	**Male**	4 (10%)
**Ethnicity**	**White**	23 (60%)
	**Black**	15 (40%)
**Pathology**	**MS**	24 (63%)
	**NMOSD**	12 (32%)
	**MOGAD**	2 (5%)
**Age at presentation**	**< 20 years**	9 (24%)
	**20–30 years**	19 (50%)
	**31–40 years**	8 (21%)
	**> 41 years**	2 (5%)

Abbreviations: MOGAD, myelin oligodendrocyte glycoprotein antibody-associated disease; MS, multiple sclerosis; NMOSD, neuromyelitis optica spectrum disorder.

In unilateral neuritis (n = 30, 79.5%), there were more patients with MS (55.5%, n = 21), NMOSD (21.5%, n = 8), and MOGAD (2.5%, n = 1). In bilateral neuritis (n = 8, 20.5%), the results were NMOSD (10.5%, n = 4), MS (7.5%, n = 3), and MOGAD (2.5%, n = 1). Analyzing each disease separately ( Table 3 ), of the patients with MS (n = 24), 87.5% had unilateral ON and 12.5% (n = 3) had bilateral presentation; out of the patients with NMOSD (n = 12), 67% (n = 8) had unilateral ON and 33% (n = 4) had bilateral presentation; MOGAD patients (n = 2) were equally divided between unilateral and bilateral ON. Other data related to imaging characteristics and other exams were not possible to obtain due to lack of data in the analyzed medical records.

**Table 3:** Laterality of the optic neuritis

Optic neuritis	MS (n = 24; 63%)	NMOSD (n = 12; 32%)	MOGAD (n = 2; 5%)
**Unilateral**	21 (87.5%)	8 (67%)	1 (50%)
**Bilateral**	3 (12.5%)	4 (33%)	1 (50%)

Abbreviations: MOGAD, myelin oligodendrocyte glycoprotein antibody-associated disease; MS, multiple sclerosis; NMOSD, neuromyelitis optica spectrum disorder.

In patients who had access to the health insurance or received private care, there was a higher percentage of MS (41.5%, n = 16), compared to patients treated in the SUS (Brazilian unified health system), which corresponds to 20.5% (n = 8). In cases of NMOSD, the opposite was observed, with 23.5% (n = 9) treated in the SUS and 7.5% (n = 3) in the private health system; all (n = 2) MOGAD patients were diagnosed in the private health system.

Regarding the time of diagnosis, observing the total number of patients (n = 38), there were equal percentages of 2.5% (n = 1) for MS and NMOSD, when evaluating the number of patients who completed diagnoses at the time of presentation of ON. There were no records of positive NMOSD and anti-MOG. Between 1 and 12 months, equal percentages of 5.2% (n = 2) were obtained for NMOSD and positive anti-MOG. There was a higher number of patients with MS, 26% (n = 10), and a lower number with NMOSD, with 2.5% (n = 1) of the cases. Between 13 and 24 months, 10.5% (n = 4) were diagnosed with MS and 2.6% (n = 1) with NMOSD; there were no records of MOGAD. Between 25 and 36 months, only NMOSD was recorded, with 2.6% (n = 1) of cases. Multiple sclerosis and MOGAD were not reported. Between 37 and 60 months, only MS was diagnosed in 2.6% (n = 1) of the patients. In those whose diagnosis took longer than 60 months, 16.5% (n = 6) of the patients were diagnosed with NMOSD and 21% (n = 8) with MS ( Table 4 ).

**Table 4:** Time until the etiological diagnosis of optic neuritis (public and private healthcare systems)

Time until diagnosis	MS (n = 24; 63%)	NMOSD (n = 12; 32%)	MOGAD (n = 2; 5%)
**Immediate**	1 (2.5%)	1 (2.5%)	0
**1–12 months**	10 (26%)	3 (8%)	2 (5%)
**13–24 months**	4 (10.5%)	1 (2.5%)	0
**25–36 months**	0	1 (2.5%)	0
**37–60 months**	1 (2.5%)	0	0
**> 60 months**	8 (21.5%)	6 (16.5%)	0

Abbreviations: MOGAD, myelin oligodendrocyte glycoprotein antibody-associated disease; MS, multiple sclerosis; NMOSD, neuromyelitis optica spectrum disorder.

Analyzing each of the pathologies individually, we found that in patients with MS (n = 24), we obtained the following results: diagnosis at the time of presentation of ON happened for 4% (n = 1) of the patients; between 1 and 12 months for 42% (n = 10); 13 to 24 months for 17% (n = 4); 25 to 36 months there was no record; 37 to 60 months for 4% (n = 1); and, finally, greater than 60 months for 33% (n = 8) of the patients. In patients with NMOSD (n = 12), we observed that 8% (n = 1) of the diagnoses were made at the time of presentation of ON; 25% (n = 3) between 1 and 12 months; 8% (n = 1) from 13 to 24 months; 8% (n = 1) from 25 to 36 months; there were no records of diagnoses between 37 and 60 months; and, finally, 50% of the patients were diagnosed 60 months after the initial picture of ON. In MOGAD patients, 100% (n = 2) were diagnosed between 1 and 12 months of ON presentation. Analyses were also constructed separately of the time to diagnosis in patients treated in the public and private health network, which can be seen in Tables 5 and 6 , respectively.

**Table 5:** Time until diagnosis in the public healthcare system

Time until diagnosis	MS (n = 8; 33%)	NMOSD (n = 9; 75%)	MOGAD (n = 0; 0%)
**Immediate**	0 (0%)	1 (14%)	0 (0%)
**1–12 months**	2 (25%)	3 (79%)	0 (0%)
**13–24 months**	2 (25%)	1 (14%)	0 (0%)
**25–36 months**	0 (0%)	1 (14%)	0 (0%)
**37–60 months**	0 (0%)	0 (0%)	0 (0%)
**> 60 months**	4 (50%)	3 (79%)	0 (0%)

Abbreviations: MOGAD, myelin oligodendrocyte glycoprotein antibody-associated disease; MS, multiple sclerosis; NMOSD, neuromyelitis optica spectrum disorder.

**Table 6:** Time until diagnosis in the private healthcare system

Time for diagnosis	MS (n = 16; 67%)	NMOSD (n = 9; 25%)	MOGAD (n = 2; 100%)
**Immediate**	1 (6.25%)	0 (0%)	0 (0%)
**1–12 months**	8 (50%)	0 (0%)	2 (100%)
**13–24 months**	2 (12.5%)	0 (0%)	0 (0%)
**25–36 months**	0 (0%)	0 (0%)	0 (0%)
**37–60 months**	1 (6.25)	0 (0%)	0 (0%)
**> 60 months**	4 (25%)	3 (100%)	0 (0%)

Abbreviations: MOGAD, myelin oligodendrocyte glycoprotein antibody-associated disease; MS, multiple sclerosis; NMOSD, neuromyelitis optica spectrum disorder.

Analyzing each disease separately ( Table 7 ) and the place where the diagnoses were made, we observed that, in the case of MS (n = 24), for which, in total, the average time for diagnosis was 19 months, the patients assisted in the public system had an average of 54 months, while for patients seen in the private sector, the average was 12 months. In the case of NMOSD, the general average was 72 months, but there was a difference between the public and private systems, with an average of 108 months being recorded for patients in the private system and 14.5 months in the public health system. All MOGAD patients came from the private system, with an average time for diagnosis of 2 months.

**Table 7:** Average time until diagnosis per disease

Disease	Time (months)
**MS**	19
**NMOSD**	72
**MOGAD**	2

Abbreviations: MOGAD, myelin oligodendrocyte glycoprotein antibody-associated disease; MS, multiple sclerosis; NMOSD, neuromyelitis optica spectrum disorder.

## DISCUSSION

The growing number of descriptions and population analyses has contributed to the understanding of the clinical and evolutionary profile of ON as an initial symptom of DDs (MS, NMOSD, and MOGAD). 6 8

Regarding gender, it was confirmed that white women are more affected by MS, with a ratio of two women for every man. 7 11 When comparing ON results between genders, it was found that women are 4 times more likely to develop it. In this study, a 23 times greater chance of occurrence in women (23:1) as an initial symptom of MS was calculated. Although consistent with the higher prevalence in women, these values were discrepant because this study analyzed ON as an initial symptom of MS, while the comparative study considered the total number of cases and ON at any time of the disease. In a study carried out in Sweden, conducted by Jin et al., the same values were attested. It was observed that men over 50 and women over 60 did not have ON. 14 The higher prevalence in women is related to the higher frequency of identification of the HLA-A3, B7, and D/DR2 genes in this gender. Furthermore, a greater risk of developing MS from ON was observed in patients who had these genes and were younger in age. Concomitantly, there is an increased risk in the 5-year period after the diagnosis of ON. 1 2 3 4

Still in conference with the ONTT, the ethnicity, sex, and age that were cited as being risk factors are: Caucasian, female (2:1), between 20 and 40 years. 6 In this study, the higher occurrence of ON cases in Caucasians (1.5:1) was investigated. In addition, the proportion between the sexes was higher in females and, although our age approach was fractionated in a divergent way, the group of participants between 20 and 40 years old was predominant. 8

As for the general average age of onset, it was 26.5 years for women and 18 years for men. In women with MS, the mean age of presentation of ON was 28 years and in the only man in the sample, with MS, it was 21 years. The results showed a slight decrease in the average age in women and a large decrease in the average age in men, in comparison with a study carried out in Finland. From it, there was controversy of results, since it was found that the general mean age of onset is 31.2 years, with early onset in women. 15

The higher prevalence of females was related to the occurrence of MS and NMOSD, but there was not enough data for a deeper analysis of MOGAD patients. A study in Japan reported a higher prevalence of MOG in females. 16 For patients with NMOSD, the mean age at the first disease outbreak is 40 years, while in this study, it was found to be 21.5 years. This discrepancy may be due to evaluating only ON cases, not considering patients who initially presented with postrema area syndrome and/or longitudinally-extensive myelitis. 17

It was demonstrated that white patients have a higher percentage than black patients of presenting ON as an initial symptom in the case of MS, which is consistent with another study that evaluated the diagnosis in general and not only regarding the initial attack. 18 Conversely, for NMOSD, the black population had a higher occurrence than the white population, which is consistent with the literature, as shown by Hor et al (2020); 17 There was no interpretation on MOGAD due to insufficient data.

It was possible to observe through the results that MS appears more frequently in unilateral compared to bilateral ON, as already observed in the literature. 19 It was also found that in cases of NMOSD, the unilateral presentation is more common than the bilateral one, whereas in the case of MOGAD patients, despite a small sample, an equal number was found between bilateral and unilateral ON.

According to the 2017 McDonald's criteria, in addition to clinical history and neurological examination, MRI and cerebrospinal fluid (CSF) analysis are necessary for earlier, faster, and safer diagnosis of MS. 20 The underdiagnosis can be questioned if access difficulty is confirmed, as most patients are in public care assistance. This study and the literature found that ON associated with MS is the most prevalent. 20

Most patients with NMOSD had their diagnosis made in the public health system. One reason for this result is that NMOSD is a less prevalent disease, rarer, and fewer doctors have expertise in it. Additionally, specialized centers for this pathology are public. The two patients with MOGAD were diagnosed in the private system, likely due to difficulty accessing antibodies in the public health system. 20 21 22

Observing the time needed to diagnose DDs from the initial symptom, such as ON ( Table 4 ), in general, it was seen that patients with MS had most of the diagnoses made between 1 and 12 months, whereas most NMOSD patients were diagnosed more than 60 months after ON presentation. In the case of patients with MOGAD, both were diagnosed between 1 and 2 months after the onset of ON.

It is interesting to analyze these data by separating patients from the public and private health systems ( Table 8 ), so we see a big difference in the time taken for diagnosis. In the case of patients with MS, the average time for diagnosis in the SUS was 54 months, compared to 12 months in the private health system. This discrepancy is thought to be due to the need for costly complementary tests (CSF analysis and MRI) and the fact that SUS patients have greater difficulty returning to consultations to show the results of the requested tests. The analysis of the mean number of months for the diagnosis of patients with NMOSD is the opposite of what was previously reported for patients with MS. In the case of patients assisted in the SUS, the average time for diagnosis was 14.5 months, and in patients in the private system, it was 108 months. Probable reasons for these data are the small number of patients treated in the private sector and the lack of professionals with knowledge of the pathology, which, in turn, is rarer than MS.

**Table 8:** Average time until diagnosis and access to the healthcare system

Disease/Assistance	Time (months)
**MS/ Public**	54
**MS/ Private**	12
**NMOSD/ Public**	14.5
**NMOSD/ Private**	108
**MOGAD/ Private**	2

Abbreviations: MOGAD, myelin oligodendrocyte glycoprotein antibody-associated disease; MS, multiple sclerosis; NMOSD, neuromyelitis optica spectrum disorder.

In conclusion, the identification of patients who presented ON as the initial symptom of some DD, including MS, NMOSD, and MOGAD, had a profile of female sex, higher prevalence of MS, age between 21 and 40 years, and predominantly white ethnicity in MS specifically. It was possible to verify that in NMOSD, the predominant ethnicity was black, totaling 75% of the sample of DDs considered, similar to the current literature. Neuromyelitis optica spectrum disorder diagnoses were made in patients younger than 30 years in 91% of cases, with the age group between 21 and 30 years being the most prevalent, unlike what was found in the literature. The most prevalent DD in patients with with ON as their initial presentation is MS, followed by NMOSD and MOGAD, respectively. The mean time for defining the diagnosis of DD from the initial symptom of ON was 19 months in cases of MS, 72 months NMOSD, and 2 months in cases of MOGAD. There are clear distinctions in time for diagnosis in public and private health systems, with MS having the shortest time for diagnosis in the private system and NMOSD in the public system, showing the need for further studies to investigate the cause of such discrepancies.
